# Trastuzumab Deruxtecan: Changing the Destiny of HER2 Expressing Solid Tumors

**DOI:** 10.3390/ijms22094774

**Published:** 2021-04-30

**Authors:** Alice Indini, Erika Rijavec, Francesco Grossi

**Affiliations:** 1 Medical Oncology Unit, Fondazione IRCCS Ca’ Granda Ospedale Maggiore Policlinico, 20122 Milan, Italy; alice.indini@gmail.com (A.I.); erika.rijavec@policlinico.mi.it (E.R.); 2 Medical Oncology Unit, Department of Medicine and Surgery, University of Insubria, ASST dei Sette Laghi, 21100 Varese, Italy

**Keywords:** trastuzumab deruxtecan, HER2, breast cancer, gastric cancer, NSCLC, colorectal cancer

## Abstract

HER2 targeted therapies have significantly improved prognosis of HER2-positive breast and gastric cancer. HER2 overexpression and mutation is the pathogenic driver in non-small cell lung cancer (NSCLC) and colorectal cancer, however, to date, there are no approved HER2-targeted therapies with these indications. Trastuzumab deruxtecan (T-DXd) is a novel HER2-directed antibody drug conjugate showing significant anti-tumor activity in heavily pre-treated HER2-positive breast and gastric cancer patients. Preliminary data have shown promising objective response rates in patients with HER2-positive NSCLC and colorectal cancer. T-DXd has an acceptable safety profile, however with concerns regarding potentially serious treatment-emergent adverse events. In this review we focus on the pharmacologic characteristics and toxicity profile of T-Dxd, and provide an update on the most recent results of clinical trials of T-DXd in solid tumors. The referenced papers were selected through a PubMed search performed on 16 March 2021 with the following searching terms: T-DXd and breast cancer, or gastric cancer, or non-small cell lung cancer (NSCLC), or colorectal cancer. Oral presentation, abstracts, and posters presented at the American Society of Clinical Oncology (ASCO, Alexandria, VA, USA) 2020 and the European Society for Medical Oncology (ESMO, Lugano, Switzerland) 2020 annual meetings were retrieved for data on T-DXd. We also overview ongoing research and data of combination therapies currently under investigation, which will impact on future therapeutic strategies. Clinicaltrials.gov was searched to identify ongoing clinical trials of T-DXd alone or in combination in solid tumors.

## 1. Introduction

HER2 targeted therapies have dramatically improved the prognosis of HER2-positive breast cancer patients in the adjuvant and neoadjuvant settings and in metastatic disease [1], and of patients with HER2-positive metastatic gastric cancer [2]. To date, several anti-HER2 drugs have been approved for the treatment of breast cancer: dual blockade with trastuzumab and pertuzumab, both anti-HER2 humanized monoclonal antibodies (mAb), in combination with chemotherapy is approved as first line treatment for metastatic disease [3]; the antibody–drug conjugate (ADC) trastuzumab emtansine (T-DM1) and the HER2 kinase inhibitor lapatinib are currently approved for further lines of treatment [4,5]. Trastuzumab in combination with chemotherapy is the standard frontline treatment for HER2-positive metastatic gastric cancer [2]. Even if less common, HER2 overexpression and mutation is the pathogenic driver even in other types of solid tumors, as non-small cell lung cancer (NSCLC) [6], colorectal [7], and biliary tract cancers [8]. However, to date there are no approved HER2-targeted therapies for HER2-expressing solid tumors with these indications.

Trastuzumab deruxtecan (T-DXd) (DS-8201; ENHERTU^®^) is a novel HER2-directed ADC which was discovered by Daiichi Sankyo Company, Ltd. (Tokyo, Japan) and jointly developed by Daiichi Sankyo Company, Ltd. in collaboration with AstraZeneca (Cambridge, UK) [9]. T-DXd has been evaluated as treatment of several HER2-expressing cancers, including HER2-positive and HER2-low breast cancer, HER2-positive gastric cancer, HER2-expressing colorectal cancer, and HER2-expressing or -mutated NSCLC [9]. T-DXd has shown significant anti-tumor activity in patients with heavily pre-treated HER2-positive metastatic breast cancer [10], thus leading to an accelerated Food and Drug Administration (FDA, Silver Spring, MD, USA) approval in 2019 [11]. Several trials are currently underway to explore T-DXd in a wide range of HER2-expressing tumors with promising preliminary results.

In this review we focus on the molecular and pharmacologic characteristics, and toxicity profile of this novel anti-HER2 compound, and provide an update on the most recent results of ongoing clinical trials of T-DXd in solid tumors. We also overview ongoing clinical research in this field and provide data of combination therapies currently under investigation, which will impact on future therapeutic strategies.

## 2. HER2 Targeting by T-DXd: Preclinical Studies

HER2 belongs to the human epidermal growth factor receptor (HER) family of four tyrosine kinases consisting of EGFR (HER1, erbB1), HER2 (erbB2, HER2/*neu*), HER3 (erbB3) and HER4 (erbB4) [12]. All together, these receptors regulate key cellular processes including proliferation, motility, and survival. The role of *HER2* in human carcinogenesis was first established when it was discovered that ~15–20% of breast cancers show HER2 amplification and overexpression, a genetic feature associated with aggressive biologic behavior and worse prognosis [13]. HER2 overexpression has a highly transforming potential, and the amplification of *HER2* locus is an early event in breast cancer carcinogenesis [14]. Approximately 15–20% of advanced gastric and gastroesophageal junction cancers, have overexpression or amplification of HER2 [2]. Mutations and amplification of ERBB2 have also been reported in ~4% of NSCLC, 2–5% of colorectal cancers, 5–20% of biliary tract tumors, and in up to 60% of salivary duct carcinomas [6,7,8,15].

**Figure 1:**
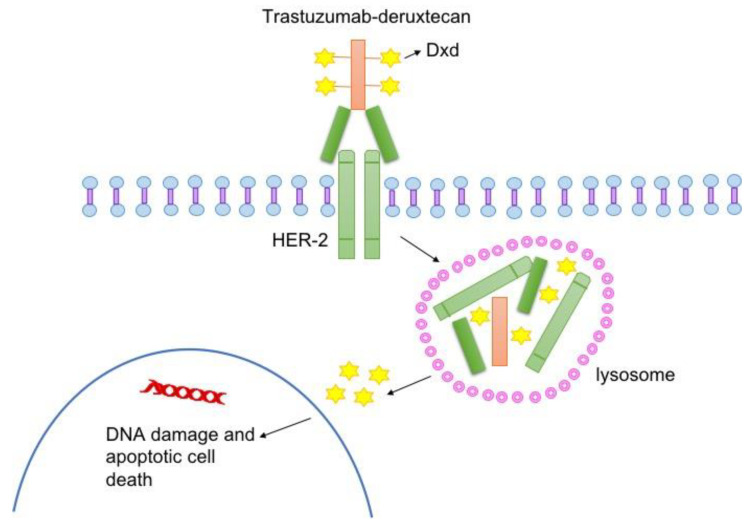
Mechanism of action of trastuzumab deruxtecan (T-DXd): following binding to HER2 on tumor cells, T-DXd undergoes internalization and intracellular linker cleavage by lysosomal ezymes. Upon release, the membrane permeable DXd enters the nucleus and causes DNA damage and apoptotic cell death.

HER2 overexpression is a strong predictive factor for response to anti-HER2 therapies, which target different regions of the HER2 protein [16]. T-DXd is a monoclonal antibody-drug conjugate (ATC code: L01XC41). T-DXd is composed of (1) an anti-HER2 humanized monoclonal immunoglobulin G1 antibody (MAAL-9001), with the same amino acid sequence as trastuzumab; (2) a cleavable maleimide tetrapeptide linker, which is selectively cleaved by cathepsins upregulated in cancer cells and in the tumor microenvironment to allow the release of the cytotoxic drug; and (3) the exatecan derivative MAAA-1181a (DXd), a DNA topoisomerase I inhibitor with a 10-fold higher inhibitory potency compared with SN-38, the active metabolite of irinotecan [17]. Upon binding to HER2, T-DXd disrupts HER2 signaling and mediates antibody-dependent cell-mediated cytotoxicity. In addition, after binding, T-DXd undergoes internalization and intracellular cleavage, resulting in release of deruxtecan. Upon release, deruxtecan causes DNA damage and apoptotic cell death. Figure 1 shows the structure and mechanism of action of T-DXd.

T-DXd was specifically designed to improve on the characteristics of other anti-HER2 ADC. Trastuzumab has been shown to modulate topoisomerase I expression in extracellular vesicles released by HER2 positive cancer cells. In this regard, combining a topoisomerase inhibitor, DXd, with trastuzumab, could have a potentially increased anti-tumor effect [18]. Compared with TDM-1, T-DXd shows a higher drug-to-antibody ratio (DAR, approximately 8 vs. 3 to 4), allowing efficient delivery to HER2 expressing tumor cells. Its cytotoxic payload has a short half-life, thus increasing the cytotoxic effect but minimizing the systemic exposure and limiting the off-target toxicity in normal cells [17]. Moreover, the payload has a high membrane permeability, which allows a cytotoxic effect on tumor cells in close proximity to targeted cells, regardless of their HER2 expression levels. This cytotoxic bystander effect has been confirmed both in in vitro and in vivo HER2-negative cells which were destroyed in presence of HER2-overexpressing cells [19]. In colorectal cancer cell lines with different levels of HER2 protein expression (without HER2/neu gene amplification), Takegawa et al., observed that HER2-overexpressing cells were sensitive to T-DXd, but not to other anti-HER2 agents [19]. This specific feature of T-DXd might potentially increase its therapeutic effect in those tumors harboring heterogeneous HER2 expression, as NSCLC and colorectal cancer [20,21].

In vitro and in vivo pharmacologic activities of T-DXd were evaluated and compared with TDM-1 in HER2-positive cell lines and patient-derived xenograft (PDX) models [17]. This study confirmed a higher membrane permeability of DXd as compared with DM1, along with high stability in plasma. The achievement of increased DAR allowed antitumor effects also on HER2-low expressing tumor models, while T-DM1 was not effective in these models. Moreover, in the PDX model, T-DXd showed potent antitumor activity against T-DM1 primary insensitive tumor cells, likely due to a higher sensitivity of T-DM1 to p-glycoprotein mediated efflux [17].

Overall, molecular characteristics of T-DXd and preclinical evidences support its role in HER2-low expressing tumors, as well as in tumors expressing HER2 protein in the absence of HER2 amplification, thus expanding the population of patients who will potentially benefit from such treatment.

## 3. Therapeutic Applications

The safety, tolerability, and activity of T-DXd in patients with advanced HER2-expressing breast and gastric and gastroesophageal junction tumors were assessed in a dose-escalation phase 1 trial, the DS8201-A-J101 (NCT02564900) [22]. In the first part of this trial, 24 patients were enrolled and received T-DXd from 0.8 to 8.0 mg/kg once every 3 weeks. Based on the results of the dose escalation phase, the maximum tolerated dose (MTD) was not reached, and the recommended phase 2 dosing (RP2D) was set on 5.4 or 6.4 mg/kg. In this small, heavily pretreated study population, T-DXd demonstrated promising antitumor activity (disease control rate (DCR) 91%), even in low HER2-expressing tumors [22].

In the dose expansion part of the study, T-DXd at the recommended doses (i.e., 5.4 mg/kg and 6.4 mg/kg once every 3 weeks) was assessed in five cohorts: advanced metastatic HER2-positive breast cancer progressed on prior TDM-1, HER2-positive gastric, or gastroesophageal junction cancer progressed on prior trastuzumab, HER2-low expressing breast cancer (i.e., immunohistochemistry (IHC) 1+ or 2+, in-situ hybridization (ISH) negative), other HER2-expressing (defined as IHC 3+, 2+, or 1+ or amplified) or HER2-mutated solid tumors [23,24]. During the dose expansion part of the trial, the MTD of 6.4 mg/kg once every 3 weeks was chosen based on the principle of highest tolerated dose without dose-limiting toxicities. Results of the dose expansion phase among HER2-positive breast and gastric cancer patients will be discussed in details in further sections [23,24]. The safety and efficacy results among HER2-positive (non-breast/non-gastric) solid tumors have recently been published [25]. Overall, 60 patients received ≥1 dose of 6.4 mg/kg T-DXd (colorectal cancer, *n* = 20; NSCLC, *n* = 18; and other, *n* = 22, including 8 salivary gland tumors; 2 breast cancers (1 HER2 low and 1 HER2 status missing per central laboratory assessment); 2 esophageal cancers; 2 endometrial cancers; 2 Paget disease; 2 biliary tract cancer; and 1 case each of pancreatic cancer, uterine cervix carcinoma, extraskeletal myxoid chondrosarcoma, and small-intestine adenocarcinoma). In this heterogeneous population, the median progression free survival (PFS) was 7.2 (95% confidence interval (CI), 4.8–11.1) months and the confirmed objective response rate (ORR) was 28.3%. Interestingly, among patients with NSCLC median PFS was 11.3 (95% CI, 8.1–14.3) months, and ORR was 72.7%. Confirmed disease response was observed in the following tumor types: HER2-expressing and mutant NSCLC, colorectal, salivary gland, biliary tract, and endometrial cancers [24]. Treatment with T-DXd resulted in an acceptable safety profile, consistent across patients’ cohorts, however with concerns regarding the onset of potentially serious treatment-emergent adverse events (TEAE) [25]. Table 1 shows the main results of major clinical trials of T-DXd.

### 3.1. Breast Cancer

T-DXd showed promising activity in the cohort of HER2-positive breast cancer patients (*n* = 115) pretreated with TDM-1 in the phase 1 trial (NCT02564900; data cut-off August 2018) [23]. Patients were treated with the recommended doses for expansion: the confirmed ORR was 59.5%, and DCR was 93.7%. The median PFS was 22.1 months, and the median overall survival (OS) was not reached. In the same phase 1 trial, T-DXd demonstrated antitumor activity also in the cohort of patients with HER2-low–expressing breast cancer refractory to standard therapies (*n* = 54) [26]: confirmed ORR was 37.0%, with median duration of response (DOR) of 10.4 months. The median PFS was 11.1 months, and the median OS was 29.4 (95% CI, 12.9 to 29.4) months [26].

Based on these positive results, the activity of T-DXd was evaluated in patients with HER2-positive metastatic breast cancer who had been previously treated with ≥2 anti-HER2 agents, including TDM-1, in the phase 2 DESTINY-Breast01 trial (NCT03248492) [10]. This two-part trial randomized patients to receive T-DXd 5.4 mg/kg (*n* = 50), 6.4 mg/kg (*n* = 48) or 7.4 mg/kg (*n* = 21) once every 3 weeks in part 1 (pharmacokinetics and dose-finding) of the trial; based on efficacy and tolerability results, the RP2D was 5.4 mg/kg once every 3 weeks (*n* = 184, of whom *n* = 50 from part 1), administered until disease progression or unacceptable treatment-related toxicity. After a median follow up of 11.0 months (data cut-off: August 2019), the confirmed ORR was 60.9%, and the confirmed DCR was 97.3%. The median time to response was 1.6 months, the median DOR 14.8 months, and the median duration of PFS was 16.4 months. The median OS was not reached, but estimated OS was 93.9% at 6 months, and 86.2% at 12 months. Results of two subgroup analyses of this trial have been presented at the 2020 American Society for Clinical Oncology (ASCO) annual meeting. The first report underlines a connection of the following variables with improved response and survival outcomes during treatment with T-DXd: hormone receptor positive status, fewer prior treatment regimens, pertuzumab given in the first or second line, and normal renal and hepatic function [27]. Notwithstanding, T-DXd demonstrated strong efficacy in all clinical subgroups analyzed. The second analysis reported data on T-DXd for patients with brain metastases at baseline and upon disease progression: T-DXd showed comparable efficacy among patients with brain metastases at baseline. Moreover, brain disease progression was noted at time of progression in only 8% of patients without brain disease involvement at the time of treatment initiation [28].

On 20 December 2019, the FDA granted accelerated approval to T-DXd for patients with unresectable or metastatic HER2-positive breast cancer who have received ≥2 prior anti-HER2-based regimens in the metastatic setting [11]. The recommended T-DXd dose is 5.4 mg/kg given once every 3 weeks until disease progression or unacceptable toxicity. On 10 December 2020, the European Medicines Agency’s (EMA, Amsterdam, The Netherlands) Committee for Medicinal Products for Human Use (CHMP) adopted a positive opinion, recommending the granting of a conditional marketing authorization for T-DXd [29].

To date, five randomized phase 3 trials of T-DXd in breast cancer patients are underway, for HER2-positive (DESTINY-Breast02, NCT03523585; DESTINY-Breast03, NCT03529110) and HER2-low (DESTINY-Breast04, NCT03734029; DESTINY-Breast06, NCT04494425) metastatic disease, and for HER2-positive primary breast cancer who do not achieve complete response after neoadjuvant therapy (DESTINY-Breast05, NCT04622319). Several other phase 1b and 2 trials are evaluating the role of T-DXd for HER2-positive and HER2-low disease in further lines of treatment, or in presence of central nervous system metastases, and in patients with triple negative breast cancer (Table 2).

### 3.2. Gastric Cancer

Overall, 44 patients with HER2-positive gastric or gastroesophageal junction cancer treated with T-DXd in the phase I trial were evaluable for safety and anti-tumor activity [24]. Eligibility criteria included disease progression on previous trastuzumab treatment. After a median follow-up of 5.5 months (data cut-off 10 August 2018), the confirmed ORR was 43.2%, and 79.5% of patients achieved DCR. The median time to response was 1.4 months, and the median DOR was 7.0 months. The median PFS was 5.6 months, and the median OS was 12.8 months. In a post-hoc subgroup analysis of 24 patients previously treated with irinotecan, ORR was 41.7% and DCR was 79.2% [24].

The randomized phase 2 trial DESTINY-Gastric01 evaluated T-DXd compared with chemotherapy in patients with HER2-positive advanced gastric or gastroesophageal junction adenocarcinoma who had progressed on at least two previous therapies, including trastuzumab (NCT03329690) [30]. In this trial, patients were randomly assigned in a 2:1 ratio to receive T-DXd 6.4 mg/kg once every 3 weeks (*n* = 125), or physicians’ choice chemotherapy (*n* = 62; 55 received irinotecan, and 7 received paclitaxel). Updated results of this trial were presented at the 2020 ASCO annual meeting (data cutoff 8 November 2019) [31]: confirmed ORR with T-DXd was 42.9% vs. 12.5% with chemotherapy, and DCR was 85.7% vs. 62.5%; median DOR was 11.3 vs. 3.9 months; median PFS was 5.6 vs. 3.5 months. OS was significantly prolonged with T-DXd, with a median OS of 12.5 vs. 8.4 months, and a 12-month OS of 52.1% with T-DXd vs. 28.9% with chemotherapy.

The efficacy of T-DXd was also evaluated in two HER2-low gastric cancer exploratory cohorts, including patients with IHC 2+/ISH− (cohort 1, *n* = 20), or IHC 1+ (cohort 2, *n* = 24) [32]. In cohort 1, confirmed ORR was 26.3%, with confirmed DCR of 89.5%. Median PFS was 4.4 months, and median OS 7.8 months, with a 12-month OS rate of 40.0%. Confirmed ORR in cohort 2 was 9.5%, and confirmed DCR was 71.4%. Median PFS was 2.8 months and median OS was 8.5 months, with a 12-month OS rate of 25.7%.

On 15 January 2021, the FDA approved T-DXd for adult patients with locally advanced or metastatic HER2-positive gastric cancer or gastroesophageal junction adenocarcinoma who have received a prior trastuzumab-based therapy regimen [33].

At the present time, two phase 2 trials in HER2-positive gastric cancer patients who progressed on prior trastuzumab are evaluating T-DXd as a monotherapy (DESTINY-Gastric02, NCT04014075) or in combination with chemotherapy and trastuzumab (DESTINY-Gastric03, NCT04379596) (Table 2).

### 3.3. NSCLC

Preclinical data suggest that the presence of activated HER2 in NSCLC cells, regardless of the addiction status to its downstream signaling pathways, can be used as a carrier to convey chemotherapeutic agents into cancer cells [34]. The clinical activity of T-DM1 in patients with ERBB2-amplified or-mutated lung cancers was confirmed in the context of a histology-agnostic phase II basket trial (NCT02675829) [35]. Indeed, the superior efficacy of T-DXd compared to TDM-1 has been demonstrated in lung tumors mouse models [34].

The DESTINY-Lung01 trial is an ongoing phase II study of T-DXd in patients with non-squamous NSCLC overexpressing HER2 or with HER2-activating mutation (NCT03505710). Preliminary data from this trial confirmed a promising clinical activity, with high ORR and durable responses among patients with HER2 mutations (*n* = 42) [36]. After a median follow-up of 8.0 months (data cut-off 25 November 2019), the median treatment duration was 7.75 months (range, 0.7–14.3 months). Confirmed ORR was 61.9%, median DOR was not reached, and DCR was 90.5%. The estimated median PFS was 14.0 mo (95% CI, 6.4–14.0 mo). Notably, 45.2% of patients had central nervous system metastases.

The four phase 2 studies and one phase 1 study in patients with HER2-positive or mutated NSCLC are ongoing: the DESTINY-Lung01 (NCT03505710), and the DESTINY-Lung02 (NCT04644237) evaluate T-DXd as monotherapy, while the DESTINY-Lung03 (NCT04686305), and the HUDSON trial (NCT03334617) investigate the combination of T-DXd with immunotherapy, chemotherapy, and novel anticancer agents.

### 3.4. Colorectal Cancer

Due to the high incidence worldwide of colorectal cancer, anti-HER2 targeted therapies have recently become an attractive matter of investigation, even if the incidence of ERBB2 genomic alterations is relatively low among patients with metastatic colorectal cancer [37]. Preliminary results have shown promising activity for the combination of trastuzumab with either lapatinib or pertuzumab in patients with heavily pretreated colorectal cancer with ERBB2 amplification [38,39].

T-DXd is currently under investigation in a phase 2 trial, in patients with HER2-expressing metastatic colorectal cancer that progressed on ≥2 prior systemic treatment regimens (DESTINY-CRC01, NCT03384940) (Table 2). In this study, patients are subdivided in 3 cohorts according to HER2 expression levels (A: HER2 IHC 3+ or IHC 2+/ISH+; B: IHC 2+/ISH−; C: IHC 1+). Preliminary data showed a remarkable activity of T-DXd in a cohort of 78 patients with heavily pretreated HER2 positive colorectal cancer (*n* = 53 in cohort A; *n* = 7 in cohort B; and *n* = 18 in cohort C) [40]. At data cutoff (9 August 2019), median treatment duration was 3.5 months (95% CI, 2.1–4.3 months). The confirmed ORR was 45.3% (95% CI, 31.6–59.6%) in cohort A; median DOR was not reached (95% CI, 4.2 months-NE). The DCR was 83.0% (95% CI, 70.2–91.9%); median PFS was 6.9 months (95% CI, 4.1 months-NE); median OS was not reached. No responses were observed in cohorts B or C.

## 4. Safety Profile

The safety of T-DXd was evaluated in a pooled analysis of 234 patients with HER2-positive breast cancer who received at least one dose of T-DXd 5.4 mg/kg in the DESTINY-Breast01 (NCT03248492), and in the phase 1 trial DS8201-A-J101 (NCT02564900) [9,10,22]. The most common adverse events (AE) (i.e., frequency ≥20%; any grade according to the common terminology criteria for adverse events (CTCAE), version 4.0)) were: nausea, fatigue, vomiting, alopecia, constipation, decreased appetite, anemia, neutropenia, diarrhea, leukopenia, cough, and thrombocytopenia. The most commonly reported (i.e., frequency ≥5%) grade 3–4 AEs were neutropenia (16%), anemia (7%), nausea (7%), fatigue (6%), and leukopenia (6%). Serious adverse events (SAE) occurred in 20% of patients, and included: interstitial lung disease (ILD), pneumonia, vomiting, nausea, cellulitis, hypokalemia, and intestinal obstruction. ILD (all grades) occurred in 9% of patients, with a median time to fist onset of 4.2 months.

Permanent treatment discontinuation due to SAEs occurred in 9% of patients, in most cases because of ILD (6% of patients). Dose interruptions and dose reductions because of treatment related toxicity were required in 33% and 18% of patients, respectively. Fatal outcomes due to AEs occurred in 4.3% of patients, with ILD being the most frequent cause (2.6%), followed by acute hepatic failure and acute kidney injury, general physical health deterioration, pneumonia and hemorrhagic shock (0.4% each).

The safety profile of T-DXd among patients with metastatic gastric cancer was consistent with that observed in the phase 1 trial, with the most common AEs (i.e., (frequency ≥30%; any grade) being neutropenia, anemia, nausea, and decreased appetite [22,24,31]. Most cases of ILD and pneumonitis in this group of patients were grade 1 or 2 in severity, and no deaths related to ILD were reported. Preliminary data from ongoing clinical trials of patients with colorectal cancer and NSCLC receiving T-DXd, show a comparable safety profile even in these patients’ population [36,40].

Overall, treatment with T-DXd appears to be relatively well tolerated, even if mature data on quality-of-life are not available yet. The incidence and seriousness of ILD is of concern, and careful monitoring for signs and symptoms of ILD is mandatory. Further data from ongoing clinical trials and post-marketing surveillance will allow the implementation of guidelines for early diagnosis and management of T-DXd-induced ILD. Based on the available safety results, T-DXd prescribing information includes a boxed warning to advise health professionals of the risk of ILD, as well as embryo-fetal toxicity [9].

## 5. Conclusions and Future Perspectives

T-DXd represents a promising therapeutic agent for HER2-expressing solid tumors. Given its outstanding success as treatment of HER2-expressing tumors, T-DXd is currently under evaluation in several clinical trials, either as a monotherapy or in combination with novel anti-cancer agents. Several phase 1 and 2 trials are investigating T-DXd in HER2-expressing solid tumors, mostly as a monotherapy but also in combination with immunotherapy (NCT03523572, NCT04042701), or PARP inhibitors (NCT04585958) (Table 2).

Available data suggest that T-DXd will become an important therapeutic weapon for the treatment of metastatic HER2-positive breast cancer patients. Based on the FDA approval, the role of T-DXd in the current treatment algorithm settles in the third line, after progression on prior trastuzumab and pertuzumab plus chemotherapy, and T-DM1. In this setting, the main competitor of T-DXd is tucatinib, a highly selective and potent anti-HER2 kinase inhibitor, which has shown promising activity in patients with metastatic breast cancer when combined with trastuzumab and capecitabine [41], specifically in those with active brain metastases [42]. At the present time, data of T-DXd efficacy among patients with brain metastases are lacking, thus suggesting that T-DXd might potentially represent the treatment of choice in previous lines for patients with extracranial only disease.

Results of ongoing clinical trials might move back T-DXd to previous lines of treatment for metastatic disease. The ongoing randomized phase 3, DESTINY-Breast03 trial is evaluating a head-to-head comparison of T-DXd and T-DM1 in patients with HER2-positive breast cancer progressed on prior trastuzumab plus chemotherapy. Trials are also evaluating T-DXd as a possible treatment strategy in the neoadjuvant and adjuvant settings. T-DXd represents an emerging treatment strategy for patients with HER2-low tumors. In this regard, Daiichi Sankyo and Roche are collaborating in the development of a HER2-low companion diagnostic test, using the VENTANA HER2 assay to identify patients with low-expressing HER2 cancers [11]. T-DXd might become a valid alternative to chemotherapy in patients with HER2-low, hormonal positive breast cancer who have exhausted available endocrine-based therapies. Nevertheless, T-DXd is under investigation in patients with triple negative breast cancer, which has historically been characterized by aggressive biology and negative prognosis.

Among patients with HER2-positive gastric cancer, T-DXd holds a promising future. To date no HER2-directed therapies other than trastuzumab have shown a significant benefit in patients with HER2-positive gastric cancer [43,44]. Moreover, the therapeutic armamentarium for the treatment of metastatic HER2-positive gastric cancer progressed on prior trastuzumab limits to single agent chemotherapy, with modest improvements in OS and low rates of disease response [45]. Indeed, T-DXd has shown impressive improvement in disease response and survival compared with chemotherapy, even in patients with HER2-low expressing tumors. Preliminary results suggest that a novel treatment strategy for patients with HER2-positive gastric cancer is approaching.

With the advent of T-DXd, HER2 might become an important therapeutic target for patients with NSCLC and colorectal cancer. Several targeted agents have been investigated as potential treatments for HER2-expressing or mutated NSCLC, including single agent afatinib [46], and trastuzumab combined with chemotherapy [47], with limited results [48]. T-DM1 achieved better response rates, ranging from 20% to 50% across different studies [35,49], however significantly lower compared to those observed with T-DXd (ORR 61.9%). Even in patients with HER2-positive colorectal cancer, single agent T-DXd has demonstrated ORR exceeding 50% compared with 30% observed with the combination of trastuzumab with either lapatinib or pertuzumab [38,39]. Overall, preliminary evidence suggest that results of ongoing clinical trials will contribute to positively modify the treatment paradigm of HER2-positive tumors in the next future.

## Figures and Tables

**Table 1:** Summary of the main results of clinical trials of T-DXd.

Trial Name, NCT Number	Type of Study	Condition(s)	Sample Size	Median Follow Up	ORR	DCR	mDOR	mPFS	mOS
DS8201-A-J101, NCT02564900	Phase 1	HER2-positive mBC pretreated with T-DM1	*n* = 115	9.9 mo	59.5%	93.7%	20.7 mo	22.1 mo	NR
		HER2-low mBC refractory to standard therapies	*n* = 54	NA	37%	87%	10.4 mo	11.1 mo	29.4 mo
		HER2-positive mGC/GEJC pretreated with ≥2 therapies, including TTZ	*n* = 44	5.5 mo	43.2%	79.5%	7.0 mo	5.6 mo	12.8 mo
		HER2-positive metastatic solid tumors ^1^	*n* = 22	9.5 mo	27.3%	81.8%	NR	11.0 mo	23.4 mo
		HER2-expressing or mutated metastatic NSCLC	*n* = 18	11.0 mo	55.6%	83.3%	9.9 mo	11.3 mo	NR
		HER2-expressing metastatic CRC	*n* = 20	3.0 mo	5%	80%	7.4 mo	4 mo	15.6 mo
DESTINY-Breast01, NCT03248492	Phase 2	HER2-positive mBC pretreated with ≥2 anti-HER2 agents	*n* = 184	11.0 mo	60.9%	97.3%	14.8 mo	16.4 mo	NR
DESTINY-Gastric01, NCT03329690	Phase 2, randomized	HER2-positive mGC/GEJC pretreated with ≥2 therapies, including TTZ	*n* = 125	NA	42.9%	85.7%	11.3 mo	5.6 mo	12.5 mo
DESTINY-Lung01, NCT03505710 *	Phase 2	HER2-expressing or mutated metastatic NSCLC	*n* = 42	8.0 mo	61.9%	90.5%	NR	14.0 mo	NA
DESTINY-CRC01, NCT03384940 *	Phase 2	HER2-expressing metastatic CRC	*n* = 78 ^2^	NA	45.3%	83%	NR	6.9 mo	NR

* Preliminary results (ongoing clinical trials). ^1^ Patients in this cohort included: *n* = 8 salivary gland tumors; *n* = 2 breast cancers (1 HER2 low and 1 HER2 status missing per central laboratory assessment); *n* = 2 esophageal cancers; *n* = 2 endometrial cancers; *n* = 2 Paget disease; *n* = 2 biliary tract cancer; *n* = 1 pancreatic cancer, *n* = 1 uterine cervix carcinoma, *n* = 1 extraskeletal myxoid chondrosarcoma, and *n* = 1 small-intestine adenocarcinoma. ^2^ Response and survival results are reported for the cohort of patients with HER2 positive (IHC 3+) tumors (*n* = 53). Abbreviations: CI, confidence interval; CRC, colorectal cancer; GEJC, gastroesophageal junction cancer; DCR, disease control rate; DOR, duration of response; IHC, immunohistochemistry; mBC, metastatic breast cancer; mGC, metastatic gastric cancer; mo, months; mOS, median overall survival; mPFS, median progression-free survival; NA, not available; NR, not reached; NSCLC, non-small cell lung cancer; ORR, objective response rate; T-DM1, trastuzumab emtansine; TTZ, trastuzumab.

**Table 2:** Overview of the main ongoing clinical trials of T-DXd in solid tumors (source: clinicaltrials.gov; and rctportal.niph.go.jp; accessed: 16 March 2021).

Trial Name, NCT Number	Type of Study	Condition(s)	Drug(s)	Estimated Sample Size	Primary Endpoint(s)
**Breast cancer**					
DESTINY-Breast02, NCT03523585	Phase 3, randomized	HER2-positive metastatic BC, progressed on prior TDM-1	T-DXd Investigator’s choice CT (TTZ/lapatinib + capecitabine)	*n* = 600	PFS by BICR
DESTINY-Breast03, NCT03529110	Phase 3, randomized	HER2-positive metastatic BC, progressed on prior TTZ + taxane	T-DXd T-DM1	*n* = 500	PFS by BICR
DESTINY-Breast04, NCT03734029	Phase 3, randomized	HER2-low metastatic BC, progressed on prior CT	T-DXd Investigator’s choice CT	*n* = 540	PFS by BICR
DESTINY-Breast05, NCT04622319	Phase 3, randomized	HER2-positive primary BC who do not achieve CR after neoadjuvant therapy	T-DXd T-DM1	*n* = 1600	IDFS
DESTINY-Breast06, NCT04494425	Phase 3, randomized	HER2-low HR-positive metastatic BC	T-DXd Investigator’s choice CT	*n* = 850	PFS
DESTINY-Breast07, NCT04538742	Phase 1b/2, randomized	HER2-positive metastatic BC, in second or later lines of treatment	T-DXd monotherapy or in combination ^1^	*n* = 350	AEs and SAEs frequency
DESTINY-Breast08, NCT04556773	Phase 1b	HER2-low metastatic BC	T-DXd + capecitabine/durvalumab+paclitaxel/capivasertib/anastrozole/fulvestrant	*n* = 185	AEs and SAEs frequency
HER2CLIMB-04, NCT04539938	Phase 2, single arm	HER2- positive metastatic BC, progressed on ≥2 prior anti-HER2-based regimens	T-DXd + tucatinib	*n* = 70	ORR
NCT04553770	Phase 2, randomized	HER2-low HR-positive early stage BC	T-DXd +/− anastrozole	*n* = 88	pCR rate
DEBBRAH, NCT04420598	Phase 2, single arm, multicohort	HER2-positive or HER2-low BC with CNS disease	T-DXd	*n* = 39	PFS, CNS ORR, OS
BEGONIA, NCT03742102	Phase 1b/2	Triple negative BC	durvalumab + T-DXd vs. durvalumab + other anti-cancer agents ^2^	*n* = 170	AEs, ORR
**Gastric cancer**					
DESTINY-Gastric02, NCT04014075	Phase 2, single arm	HER2-positive gastric cancer, progressed on prior TTZ	T-DXd	*n* = 74	ORR by BICR
DESTINY-Gastric03, NCT04379596	Phase 1b/2, randomized	HER2-positive gastric cancer, progressed on prior TTZ	T-DXd monotherapy or in combination ^3^ TTZ, 5FU/capecitabine + cisplatin/oxaliplatin	*n* = 220	AEs and SAEs frequency, ORR
**NSCLC**					
DESTINY-Lung01, NCT03505710	Phase 2, single arm	HER2-expressing or mutated NSCLC	T-DXd	*n* = 170	ORR by BICR
DESTINY-Lung02, NCT04644237	Phase 2, randomized	HER2-mutated metastatic NSCLC	T-DXd 6.4 mg/kg q3w T-DXd 5.4 mg/kg q3w	*n* = 150	ORR by BICR
DESTINY-Lung03, NCT04686305	Phase 1b	HER2-positive treatment naive non-squamous NSCLC	T-DXd + durvalumab +/−CDDP/CBDCA or pemetrexed	*n* = 120	AEs and SAEs frequency
HUDSON, NCT03334617	Phase 2, biomarker directed, umbrella study	NSCLC, progressed on prior anti-PD1/PD-L1 therapy	durvalumab + T-DXd vs. durvalumab + other novel anti-cancer agents ^4^	*n* = 410	ORR
**Miscellaneous**					
DESTINY-CRC01, NCT03384940	Phase 2	HER2-expressing colorectal cancer, progressed on ≥2 prior lines of CT	T-DXd	*n* = 90	ORR
NCT04616560	Phase 2, single arm	Newly diagnosed or recurrent HER2-positive osteosarcoma ^5^	T-DXd	*n* = 77	% of event-free patients at 24 weeks
DESTINY-PanTumor01, NCT04639219	Phase 2, single arm	HER2-expressing metastatic solid tumors	T-DXd	*n* = 100	ORR by BICR
DESTINY-PanTumor02, NCT04482309	Phase 2, single arm	HER2-expressing metastatic solid tumors ^6^	T-DXd	*n* = 280	ORR
HERB, JMA-IIA00423	Phase 2, single arm	HER2-expressing biliary tract cancer	T-DXd	*n* = 32	ORR
NCT03523572	Phase 1b	HER2-expressing BC, urothelial cancer	T-DXd + nivolumab	*n* = 99	AEs frequency, ORR
NCT04585958	Phase 1	Uterine serous carcinoma, HER2-positive or -expressing solid tumors	T-DXd + olaparib	*n* = 51	MTD, AEs frequency
NCT04042701	Phase 1b	HER2-positive BC, HER2-expressing or mutated NSCLC	T-DXd + pembrolizumab	*n* = 115	MTD, ORR

^1^ Combination therapies include: durvalumab, pertuzumab, paclitaxel, durvalumab and paclitaxel. ^2^ Anti-cancer agents include paclitaxel monotherapy, or in combination with capivasertib, or oleclumab. ^3^ Combination therapies include: 5FU, capecitabine, durvalumab, 5FU/capecitabine + oxaliplatin, 5FU/capecitabine + durvalumab. ^4^ Novel anti-cancer agents include: olaparib, AZD9150, AZD6738, vistusertib (AZD2014), oleclumab, cediranib, ceralasertib. ^5^ Patients with confirmed HER2 expression of >10% of tumor cells are eligible for enrolment in this trial. ^6^ This trial includes 7 tumor-specific cohorts: urothelial bladder cancer, biliary tract cancer, cervical cancer, endometrial cancer, ovarian cancer, pancreatic cancer, and rare tumors. Abbreviations: AEs, adverse events; BC, breast cancer; BICR, blinded independent central review; CBDCA, carboplatin; CDDP, cisplatin; CNS, central nervous system; CR, complete response; CT, chemotherapy; HR, hormone receptors; IDFS, invasive disease-free survival; MTD, maximum tolerated dose; NSCLC, non-small cell lung cancer; ORR, objective response rate; pCR, pathologic complete response; PFS, progression-free survival; q3w, once every 3 weeks; SAEs, serious adverse events; T-DM1, trastuzumab emtansine; T-DXd, trastuzumab deruxtecan; TTZ, trastuzumab; 5-FU, 5-fluorouracile.

## Data Availability

No new data were created or analyzed in this study. Data sharing is not applicable to this article.

## References

[1] Larionov A.A. (2018). Current therapies for human epidermal growth factor receptor 2-positive metastatic breast cancer patients. Front. Oncol..

[2] Bang Y.J., Van Cutsem E., Feyereislova A., Chung H.C., Shen L., Sawaki A., Lordick F., Ohtsu A., Omuro Y., Satoh T. (2010). Trastuzumab in combination with chemotherapy versus chemotherapy alone for treatment of HER2-positive advanced gastric or gastro-oesophageal junction cancer (ToGA): A phase 3, open-label, randomised controlled trial. Lancet.

[3] Swain S.M., Baselga J., Kim S.B., Ro J., Semiglazov V., Campone M., Ciruelos E., Ferrero J.M., Schneeweiss A., Heeson S. (2015). Pertuzumab, trastuzumab, and docetaxel in HER2-positive metastatic breast cancer. N. Engl. J. Med..

[4] Verma S., Miles D., Gianni L., Krop I.E., Welslau M., Baselga J., Pegram M., Oh D.-Y., Diéras V., Guardino E. (2012). Trastuzumab emtansine for HER2-positive advanced breast cancer. N. Engl. J. Med..

[5] Geyer C.E., Forster J., Lindquist D., Chan S., Romieu C.G., Pienkowski T., Jagiello-Gruszfeld A., Crown J., Chan A., Kaufman B. (2006). Lapatinib plus capecitabine for HER2-positive advanced breast cancer. N. Engl. J. Med..

[6] Mazieres J., Peters S., Lepage B., Cortot A.B., Barlesi F., Beau-Faller M., Besse B., Blons H., Mansuet-Lupo A., Urban T. (2013). Lung cancer that harbors an HER2 mutation: Epidemiologic characteristics and therapeutic perspectives. J. Clin. Oncol..

[7] Siena S., Sartore-Bianchi A., Marsoni S., Hurwitz H.I., McCall S.J., Penault-Llorca F., Srock S., Bardelli A., Trusolino L. (2018). Targeting the human epidermal growth factor receptor 2 (HER2) oncogene in colorectal cancer. Ann. Oncol..

[8] Nam A.R., Kim J.W., Cha Y., Ha H., Park J.E., Bang J.H., Jin M.H., Lee K.H., Kim T.Y., Han S.W. (2016). Therapeutic implication of HER2 in advanced biliary tract cancer. Oncotarget.

[9] Daiichi Sankyo Inc. (2019). Enhertu (Fam-Trastuzumab Deruxtecan-Nxki): US Prescribing Information. https://www.accessdata.fda.gov/drugsatfdadocs/label/2019/761139s000lbl.pdf.

[10] Modi S., Saura C., Yamashita T., Park Y.H., Kim S.B., Tamura K., Andre F., Iwata H., Ito Y., Tsurutani J. (2020). Trastuzumab Deruxtecan in Previously Treated HER2-Positive Breast Cancer. N. Engl. J. Med..

[11] Keam S.J. (2020). Trastuzumab Deruxtecan: First Approval. Drugs.

[12] Moasser M.M. (2007). Targeting the function of the HER2 oncogene in human cancer therapeutics. Oncogene.

[13] Slamon D.J., Leyland-Jones B., Shak S., Fuchs H., Paton V., Bajamonde A., Fleming T., Eiermann W., Wolter J., Pegram M. (2001). Use of chemotherapy plus a monoclonal antibody against HER2 for metastatic breast cancer that overexpresses HER2. N. Engl. J. Med..

[14] Arteaga C.L., Engelman J.A. (2014). ERBB receptors: From oncogene discovery to basic science to mechanism-based cancer therapeutics. Cancer Cell.

[15] Di Villeneuve L., Souza I.L., Tolentino F.D.S., Ferrarotto R., Schvartsman G. (2020). Salivary Gland Carcinoma: Novel Targets to Overcome Treatment Resistance in Advanced Disease. Front Oncol..

[16] Tesch M.E., Gelmon K.A. (2020). Targeting HER2 in Breast Cancer: Latest Developments on Treatment Sequencing and the Introduction of Biosimilars. Drugs.

[17] Ogitani Y., Aida T., Hagihara K., Yamaguchi J., Ishii C., Harada N., Soma M., Okamoto H., Oitate M., Arakawa S. (2016). DS-8201a, a novel HER2-targeting ADC with a novel DNA topoisomerase I inhibitor, demonstrates a promising antitumor efficacy with differentiation from T-DM1. Clin. Cancer Res..

[18] Marconi S., Santamaria S., Bartolucci M., Stigliani S., Aiello C., Gagliani M.C., Bellese G., Petretto A., Cortese K., Castagnola P. (2021). Tratuzumab modulates the Protein Cargo of Extracellular Vesicles Released by ERBB2+ Breast Cancer Cells. Membranes.

[19] Takegawa N., Tsurutani J., Kawakami H., Yonesaka K., Kato R., Haratani K., Hayashi H., Takeda M., Nonagase Y., Maenishi O. (2019). [Fam-] trastuzumab deruxtecan, antitumor activity is dependent on HER2 expression level rather than on HER2 amplification. Int. J. Cancer.

[20] Grob T.J., Kannengiesser I., Tsourlakis M.C., Atanackovic D., Koenig A.M., Vashist Y.K., Klose H., Marx A.H., Koops S., Simon R. (2012). Heterogeneity of ERBB2 amplification in adenocarcinoma, squamous cell carcinoma and large cell undifferentiated carcinoma of the lung. Mod. Pathol..

[21] Marx A.H., Burandt E.C., Choschzick M., Simon R., Yekebas E., Kaifi J.T., Mirlacher M., Atanackovic D., Bokemeyer C., Fiedler W. (2010). Heterogenous high-level HER-2 amplification in a small subset of colorectal cancers. Hum. Pathol..

[22] Doi T., Shitara K., Naito Y., Shimomura A., Fujiwara Y., Yonemori K., Shimizu C., Shimoi T., Kuboki Y., Matsubara N. (2017). Safety, pharmacokinetics, and antitumour activity of trastuzumab deruxtecan (DS-8201), a HER2-targeting antibody-drug conjugate, in patients with advanced breast and gastric or gastro-oesophageal tumours: A phase 1 dose-escalation study. Lancet Oncol..

[23] Tamura K., Tsurutani J., Takahashi S., Iwata H., Krop I.E., Redfern C., Sagara Y., Doi T., Park H., Murthy R.K. (2019). Trastuzumab deruxtecan (DS-8201a) in patients with advanced HER2-positive breast cancer previously treated with trastuzumab emtansine: A dose-expansion, phase 1 study. Lancet Oncol..

[24] Shitara K., Iwata H., Takahashi S., Tamura K., Park H., Modi S., Tsurutani J., Kadowaki S., Yamaguchi K., Iwasa S. (2019). Trastuzumab deruxtecan (DS-8201a) in patients with advanced HER2-positive gastric cancer: A dose-expansion, phase 1 study. Lancet Oncol..

[25] Tsurutani J., Iwata H., Krop I., Jänne P.A., Doi T., Takahashi S., Park H., Redfern C., Tamura K., Wise-Draper T.M. (2020). Targeting HER2 with Trastuzumab Deruxtecan: A Dose-Expansion, Phase I Study in Multiple Advanced Solid Tumors. Cancer Discov..

[26] Modi S., Park H., Murthy R.K., Iwata H., Tamura K., Tsurutani J., Moreno-Aspitia A., Doi T., Sagara Y., Redfern C. (2020). Antitumor Activity and Safety of Trastuzumab Deruxtecan in Patients With HER2-Low-Expressing Advanced Breast Cancer: Results From a Phase Ib Study. J. Clin. Oncol..

[27] Modi S., Andre F., Krop I.E., Park Y.H., Kim S.B., Tamura K., Andre F., Iwata H., Ito Y., Tsurutani J. (2020). Trastuzumab deruxtecan for HER2-positive metastatic breast cancer: DESTINY-Breast01 subgroup analysis. J. Clin. Oncol..

[28] Jerusalem G., Park Y.H., Yamashita T., Hurvitz S.A., Chen S., Cathcart J., Lee C., Perrin C. (2020). CNS metastases in HER2-positive metastatic breast cancer treated with trastuzumab deruxtecan: DESTINY-Breast01 subgroup analyses. Ann. Oncol..

[29] European Medicines Agency (EMA) CHMP Summary of Positive Opinion for Enhertu. https://www.ema.europa.eu/en/medicines/human/summaries-opinion/enhertu.

[30] Shitara K., Bang Y.J., Iwasa S., Sugimoto N., Ryu M., Sakai D., Chung H.C., Kawakami H., Yabusaki H., Lee J. (2020). Trastuzumab Deruxtecan in Previously Treated HER2-Positive Gastric Cancer. N. Engl. J. Med..

[31] Shitara K., Bang Y.J., Iwasa S., Sugimoto N., Ryu M., Sakai D., Chung H.C., Kawakami H., Yabusaki H., Lee J. (2020). Trastuzumab deruxtecan (T-DXd; DS-8201) in patients with HER2-positive advanced gastric or gastroesophageal junction (GEJ) adenocarcinoma: A randomized, phase II.; multicenter, open-label study (DESTINY-Gastric01). J. Clin. Oncol..

[32] Yamaguchi K., Bang Y., Iwasa S., Sugimoto N., Ryu M., Chung H.C., Kawakami H., Yabusaki H., Lee J., Saito K. (2020). Trastuzumab deruxtecan (T-DXd; DS-8201) in patients with HER2-low, advanced gastric or gastroesophageal junction (GEJ) adenocarcinoma: Results of the exploratory cohorts in the phase II.; multicenter, open-label DESTINY-Gastric01 study. Ann. Oncol..

[33] FDA Approves Fam-Trastuzumab Deruxtecan-Nxki for HER2-Positive Gastric Adenocarcinomas [News Release]. Published January 15th, 2021. https://fda.gov/drugs/drug-approvals-and-databases/fda-approves-fam-trastuzumab-deruxtecan-nxki-her2-positive-gastric-adenocarcinomas.

[34] Li B.T., Michelini F., Misale S., Cocco E., Baldino L., Cai Y., Shifman S., Tu H.Y., Myers M.L., Xu C. (2020). HER2-Mediated Internalization of Cytotoxic Agents in ERBB2 Amplified or Mutant Lung Cancers. Cancer Discov..

[35] Li B.T., Shen R., Buonocore D., Olah Z.T., Ni A., Ginsberg M.S., Ulaner G.A., Offin M., Feldman D., Hembrough T. (2018). Adotrastuzumab emtansine for patients with HER2-mutant lung cancers: Results from a phase II Basket Trial. J. Clin. Oncol..

[36] Smit E.F., Nakagawa K., Nagasaka M., Felip E., Goto Y., Li B.T., Pacheco J.M., Murakami H., Barlesi F., Saltos A.N. (2020). Trastuzumab deruxtecan (T-DXd; DS-8201) in patients with HER2-mutated metastatic non-small cell lung cancer (NSCLC): Interim results of DESTINY-Lung01. J. Clin. Oncol..

[37] Ross J.S., Fakih M., Ali S.M., Elvin J.A., Schrock A.B., Suh J., Vergilio J.A., Ramkissoon S., Severson E., Daniel S. (2018). Targeting HER2 in colorectal cancer: The landscape of amplification and short variant mutations in ERBB2 and ERBB3. Cancer.

[38] Sartore-Bianchi A., Trusolino L., Martino C., Bencardino K., Lonardi S., Bergamo F., Zagonel V., Leone F., Depetris I., Martinelli E. (2016). Dual-targeted therapy with trastuzumab and lapatinib in treatment-refractory, KRAS codon 12/13 wild-type, HER2-positive metastatic colorectal cancer (HERACLES): A proof-of-concept, multicentre, open-label, phase 2 trial. Lancet Oncol..

[39] Meric-Bernstam F., Hurwitz H., Raghav K.P.S., McWilliams R.R., Fakih M., VanderWalde A., Swanton C., Kurzrock R., Burris H., Sweeney C. (2019). Pertuzumab plus trastuzumab for HER2-amplified metastatic colorectal cancer (MyPathway): An updated report from a multicentre, open-label, phase 2a, multiple basket study. Lancet Oncol..

[40] Siena S., Di Bartolomeo M., Raghav K.P.S., Masuishi T., Loupakis F., Kawakami H., Yamaguchi K., Nishina T., Fakih M., Elez E. (2020). A phase II, multicenter, open-label study of trastuzumab deruxtecan (T-DXd; DS-8201) in patients (pts) with HER2-expressing metastatic colorectal cancer (mCRC): DESTINY-CRC01. J. Clin. Oncol..

[41] Murthy R.K., Loi S., Okines A., Paplomata E., Hamilton E., Hurvitz S.A., Lin N.U., Borges V., Abramson V., Anders C. (2020). Tucatinib, Trastuzumab, and Capecitabine for HER2-Positive Metastatic Breast Cancer. N. Engl. J. Med..

[42] Lin N.U., Borges V., Anders C., Murthy R.K., Paplomata E., Hamilton E., Hurvitz S., Loi S., Okines A., Abramson V. (2020). Intracranial Efficacy and Survival With Tucatinib Plus Trastuzumab and Capecitabine for Previously Treated HER2-Positive Breast Cancer With Brain Metastases in the HER2CLIMB Trial. J. Clin. Oncol..

[43] Tabernero J., Hoff P.M., Shen L., Ohtsu A., Shah M.A., Cheng K., Song C., Wu H., Eng-Wong J., Kim K. (2018). Pertuzumab plus trastuzumab and chemotherapy for HER2-positive metastatic gastric or gastro-oesophageal junction cancer (JACOB): Final analysis of a double-blind, randomised, placebo-controlled phase 3 study. Lancet Oncol..

[44] Satoh T., Xu R.-H., Chung H.C., Sun G.P., Doi T., Xu J.M., Tsuji A., Omuro Y., Li J., Wang J.W. (2014). Lapatinib plus paclitaxel versus paclitaxel alone in the second-line treatment of HER2-amplified advanced gastric cancer in Asian populations: TyTAN-a randomized, phase III study. J. Clin. Oncol..

[45] Shitara K., Doi T., Dvorkin M., Mansoor W., Arkenau H.T., Prokharau A., Alsina M., Ghidini M., Faustino C., Gorbunova V. (2018). Trifluridine/tipiracil versus placebo in patients with heavily pretreated metastatic gastric cancer (TAGS): A randomised, double-blind, placebo-controlled, phase 3 trial. Lancet Oncol..

[46] Dziadziuszko R., Smit E.F., Dafni U., Wolf J., Wasąg B., Biernat W., Finn S.P., Kammler R., Tsourti Z., Rabaglio M. (2019). Afatinib in NSCLC With HER2 Mutations: Results of the Prospective, Open-Label Phase II NICHE Trial of European Thoracic Oncology Platform (ETOP). J. Thorac. Oncol..

[47] de Langen A.J., Jebbink M., Hashemi S.M.S., Kuiper J.L., de Bruin-Visser J., Monkhorst K., Thunnissen E., Smit E.F. (2018). Trastuzumab and paclitaxel in patients with EGFR mutated NSCLC that express HER2 after progression on EGFR TKI treatment. Br. J. Cancer.

[48] Mazières J., Barlesi F., Filleron T., Besse B., Monnet I., Beau-Faller M., Peters S., Dansin E., Früh M., Pless M. (2016). Lung cancer patients with HER2 mutations treated with chemotherapy and HER2-targeted drugs: Results from the European EUHER2 cohort. Ann. Oncol..

[49] Peters S., Stahel R., Bubendorf L., Bonomi P., Villegas A., Kowalski D.M., Baik C.S., Isla D., Carpeno J.C., Garrido P. (2019). Trastuzumab Emtansine (T-DM1) in Patients with Previously Treated HER2-Overexpressing Metastatic Non-Small Cell Lung Cancer: Efficacy, Safety, and Biomarkers. Clin. Cancer Res..

